# Hypocapnia is a biological marker for orthostatic intolerance in some patients with chronic fatigue syndrome

**DOI:** 10.1186/1476-5918-6-2

**Published:** 2007-01-30

**Authors:** Benjamin H Natelson, Roxann Intriligator, Neil S Cherniack, Helena K Chandler, Julian M Stewart

**Affiliations:** 1 Department of Neurosciences, UMDNJ-New Jersey Medical School, Newark NJ, USA; 2 Department of Medicine, UMDNJ-New Jersey Medical School, Newark NJ, USA; 3 Department of Pediatrics, New York Medical College, Valhalla, NY, USA

## Abstract

**Context:**

Patients with chronic fatigue syndrome and those with orthostatic intolerance share many symptoms, yet questions exist as to whether CFS patients have physiological evidence of orthostatic intolerance.

**Objective:**

To determine if some CFS patients have increased rates of orthostatic hypotension, hypertension, tachycardia, or hypocapnia relative to age-matched controls.

**Design:**

Assess blood pressure, heart rate, respiratory rate, end tidal CO2 and visual analog scales for orthostatic symptoms when supine and when standing for 8 minutes without moving legs.

**Setting:**

Referral practice and research center.

**Participants:**

60 women and 15 men with CFS and 36 women and 4 men serving as age matched controls with analyses confined to 62 patients and 35 controls showing either normal orthostatic testing or a physiological abnormal test.

**Main outcome measures:**

Orthostatic tachycardia; orthostatic hypotension; orthostatic hypertension; orthostatic hypocapnia or combinations thereof.

**Results:**

CFS patients had higher rates of abnormal tests than controls (53% vs 20%, p < .002), but rates of orthostatic tachycardia, orthostatic hypotension, and orthostatic hypertension did not differ significantly between patients and controls (11.3% vs 5.7%, 6.5% vs 2.9%, 19.4% vs 11.4%, respectively). In contrast, rates of orthostatic hypocapnia were significantly higher in CFS than in controls (20.6% vs 2.9%, p < .02). This CFS group reported significantly more feelings of illness and shortness of breath than either controls or CFS patients with normal physiological tests.

**Conclusion:**

A substantial number of CFS patients have orthostatic intolerance in the form of orthostatic hypocapnia. This allows subgrouping of patients with CFS and thus reduces patient pool heterogeneity engendered by use of a clinical case definition.

## Background

Chronic fatigue syndrome (CFS) is an ailment characterized by medically unexplained fatigue, severe enough to produce a substantial decrease in activity plus infectious, rheumatological and neuro-psychiatric symptoms. Orthostatic intolerance (OI) is defined by medically unexplained symptoms of lightheadedness, fatigue, neurocognitive deficits, nausea, abdominal pain, and shortness of breath when upright and improved by recumbency; patients with OI often have a chronic problem with fatigue even when not standing [1]. CFS patients commonly complain of symptom worsening during standing [2], and one early study reported that 22 of 23 CFS patients reported symptom worsening during orthostatic challenge. [3]. This association led to the hypothesis that some CFS patients had orthostatic intolerance which could be identified, quantified, and specifically treated.

Evaluation for OI in CFS has usually focused on abnormalities of heart rate and blood pressure control. An early report noted a high rate of delayed, neurally mediated hypotension (NMH) during upright tilt table testing of CFS patients. [3]. Although there is evidence in support of more NMH in CFS patients than healthy controls [4,5], two carefully controlled studies matching patients to controls found no difference in prevalence of this orthostatic syndrome [6,7]. A second symptomatic physiological abnormality occurring in CFS patients was reported to be orthostatic tachycardia. However, some groups reported increased rates of this physiological marker of orthostatic intolerance in CFS [8,9] while others did not [10]. One recent population-based study found no evidence for OI in CFS [11]. Thus, the existence of OI in CFS remains controversial.

A recent report noted that cardiovascular measures of OI in patients with CFS were often accompanied by hypocapnia, a pulmonary manifestation of OI where blood carbon dioxide is at lower levels than normal [9]. Since respiratory indices had not previously been assessed during orthostatic challenge, this report led us to hypothesize that the primary manifestation of OI in CFS might be orthostatic hypocapnia. To investigate this hypothesis, we performed standing tests in CFS patients and in age- and sex-matched healthy volunteers.

## Methods

The subjects were 75 patients (60 women and 15 men) fulfilling the 1994 case definition for CFS. [12]. Thus all these patients reported having new onset of fatigue that was severe enough to produce a substantial decrease in activity as well as having problems with at least four of eight infectious, rheumatological or neuropsychiatric symptoms. No medical explanation for the fatigue could be found with a set of rule-out blood tests including thyroid and liver panels, CBC, Lyme titer, ANA, and rheumatoid factor. The patients came either from a tertiary care practice devoted to medically unexplained illnesses or as volunteers responding to media reports on our research; they were evaluated regardless of medication regimen. Because earlier work had suggested an association between CFS illness severity and cardiac function. [13], patients were stratified into "severe" and "not severe" groups (30 and 45, respectively). "Severe CFS" was defined as those patients also fulfilling the more demanding 1988 case definition for CFS [14] and endorsing at least seven of the minor symptoms as producing substantial, severe or very severe problems for the patient in the month prior to intake (i.e., ≥ 3 on zero to five Likert scales). Subjects also included 40 age matched controls reporting themselves to be in excellent or good health and not taking any medications other than birth control pills (36 women and 4 men). The controls came from a data base of individuals interested in participating in research or via recruitment by research staff.

After giving informed consent, subjects filled out a questionnaire to assess current mood (Centers for Epidemiological Study-Depression. [15]) and were instrumented with a blood pressure cuff (OMRON HEM-711AC IntelliSense Automatic Blood Pressure Monitor) to allow automatic determination of blood pressure and heart rate and a nasal cannula to allow automatic determination of respiratory rate and end tidal CO2 (Oridion Microstream). Subjects were instructed in the use of visual analog scales to indicate their levels of dizziness, anxiety, shortness of breath, and of feeling ill (10 cm horizontal lines ranging from "not at all" to "as ____ as I can imagine." They were allowed to lie undisturbed for 10 minutes and then each of the above variables were recorded twice – one minute apart – with the subject in the supine position. Then, subjects were asked to stand with their feet about 8 inches from a wall; they were then told to lean back, touching only their upper back to the wall and not allowing movement of their legs for 8 minutes. This is a variant of a test used by NASA researchers to test for OI [16]; it reduces muscular influences on venous return, a major cause of variability in orthostatic testing. Heart rate, respiratory rate, blood pressure and eTCO2 as well as self report data of symptom severity were collected every minute while leaning upright.

Orthostatic tachycardia was defined as (a) more than one standing reading showing an increase from baseline of ≥ 30 beats per minute or an absolute rate of 120 beats per minute or (b) one such reading prior to subjects' being unable to tolerate further standing. Orthostatic hypertension was defined as (a) more than one standing systolic reading of ≥ 140 mmHg or diastolic reading of ≥ 90 mmHg or (b) one such systolic or diastolic reading prior to subjects' being unable to tolerate further standing. Orthostatic hypotension was defined as (a) more than one standing reading showing a drop of blood pressure of ≥ 20 mmHg systolic or 10 mmHg diastolic or (b) one such reading prior to subjects' being unable to tolerate further standing. Orthostatic hypocapnia was defined as (a) more than one standing reading of ≤ 30 mmHg eTCO2 or (b) one such reading prior to subjects' being unable to tolerate further standing. The presence of orthostatic tachycardia, orthostatic hypotension, orthostatic hypertension, or orthostatic hypocapnia defined an abnormal standing test.

### Data analysis and statistics

Statistical analysis to determine differences of "count" data between CFS and controls used Fisher's tests, and differences of continuous data used one-way ANOVAs with subsequent Bonferroni tests; when p values are given for individual post-hoc comparisons, the overall F value was significant to < .05. For data with repeated measures, hierarchical linear modeling (SPSS, Mixed) was done to assess differences from mean supine to standing values among groups. While we had a complete data set for physiological measures, we did not have self report data from 25 patients and 5 controls as we only began collecting these data after we realized they would be needed to evaluate the possibility that anxiety or depressed mood might explain our findings. Results are presented as means ± s.e.m. unless otherwise specified.

## Results

Because of the possibility that one abnormal reading while standing might have been an erroneous reading, we dropped data from subjects with only one abnormal blood pressure or one eTCO2 value in the absence of orthostatic symptoms (4 controls and 5 CFS and 1 control and 3 CFS respectively). In addition, we excluded from further analysis data from 3 CFS patients with baseline end tidal C02 values ≥ 30 mmHg because they appeared to be chronic hyperventilators [17]. Including the data from all these subjects would not have changed the overall results of this study. Finally two CFS patients on treatment for hypertension developed orthostatic hypotension, and so their data were also dropped. Following these exclusions, we analyzed the data from 62 CFS patients and 35 controls.

**Figure 1:**
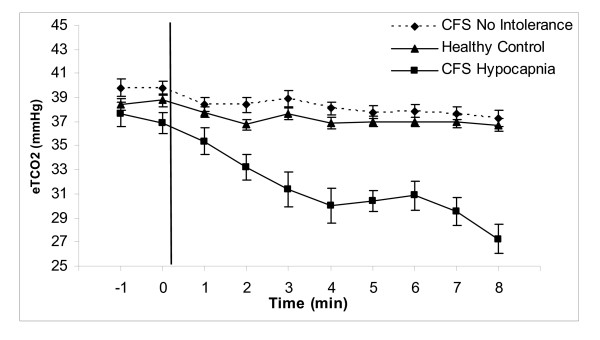
End tidal CO2 (mmHg) and time (min) before and after upright leaning. Data are presented as means ± sem over time. Subjects were in the supine position at -1 and 0 and then stood up, leaning their upper backs against a wall without moving their legs. Both controls and CFS patients without orthostatic intolerance showed a small decline in eTCO2 over time. In contrast, the fall in those with orthostatic hypocapnia was substantial.

There was no significant difference in age between patients and controls (43.3 ± 10.5 [sd] years and 40.4 ± 7.9). Significantly more CFS patients than controls fulfilled our criteria for abnormal standing tests (53% vs 20%, p < .002). For both groups, abnormalities were mostly confined to one parameter – heart rate, blood pressure or end tidal CO2 (see Table 1). Rates of orthostatic tachycardia, orthostatic hypotension, and orthostatic hypertension did not differ significantly between patients and controls (11.3% vs 5.7%, 6.5% vs 2.9%, 19.4% vs 11.4%, respectively; note that some subjects had more than one form of OI). However rates of orthostatic hypocapnia were significantly higher in CFS than in controls (20.6% vs 2.9%, p < .02). The first occurrence of a hypocapnic value occurred in the first 3 minutes of standing for 8 of the 13 subjects. However, the magnitude of hypocapnia increased over time (see Figure 1).

**Table 1:** Rates of Normal and Different Abnormal Standing Tests

	CFS	Controls
Normal	30	28
Orthostatic tachycardia (OT) alone	5	1
Orthostatic hypertension (HT) alone	9	4
Orthostatic hypertension (HT) plus OT	1	0
Orthostatic hypotension (ht) alone	4	1
Orthostatic hypocapnia alone	11	0
Orthostatic hypocapnia plus OT	0	1
Orthostatic hypocapnia plus HT	1	0
Orthostatic hypocapnia plus HT plus OT	1	0

Total Normal/Abnormal	30/32	28/7

In an effort to evaluate possible variables producing orthostatic hypocapnia, we did a post-hoc analysis confined to CFS patients with orthostatic hypocapnia (n = 13) with two comparison groups – CFS patients with normal physiological responses to orthostatic challenge (n = 30) and healthy subjects with normal physiological responses to orthostatic challenge (n = 28).

There was no difference in rates of "severe CFS" between patients with orthostatic hypocapnia and patients with no orthostatic intolerance (38% vs 30%). There was no difference in the change in respiratory rates from supine to standing among groups; however both CFS groups tended to breath slower while supine than controls (orthostatic hypocapnia: 15.4 ± 1.4; no intolerance: 16.1 ± 0.6; controls: 18.5 ± 0.6; p = .052 and .06 for each comparison). There were no differences among the 3 groups for supine systolic/diastolic blood pressure or for heart rate while supine or for the magnitude of change when standing. During orthostatic challenge, end tidal CO2 values showed a small decline over time for the CFS group without orthostatic intolerance and the controls (F_7,185.5 _= 2.89, p < .001); this effect of orthostatic challenge on normals has been previously reported [18].

Anxiety and illness ratings in the supine position were higher in both CFS groups than in the controls (p < .04 for comparisons on anxiety and < .001 on illness). Ratings of shortness of breath and dizziness in the supine position did not differ among groups.

Magnitude of change in anxiety did not differ among groups going from supine to standing. Magnitude of change in feeling ill ratings going from supine to standing increased for the CFS group with orthostatic hypocapnia but not for the other CFS group or the controls (F_ [Int]7,60.5 _= 4.14; p < .001). Magnitude of change in shortness of breath going from supine to standing was significantly greater for the CFS group with orthostatic hypocapnia than the CFS group without orthostatic intolerance (F_1,30.4 _= 4.44, p < .05). Both CFS groups reported a greater increase in shortness of breath while standing compared to controls (p < .005 for both comparisons). In terms of increases in dizziness ratings going from supine to standing, it was the CFS group without orthostatic abnormalities that was higher than controls (F_1,47.3 _= 14.2, p < .001) with the CFS group with hypocapnia being intermediary.

There was no significant difference in depressed mood between the 2 CFS groups as assessed by the CES-D, but both were significantly higher than controls (p < .001; medians for those with orthostatic hypocapnia, no orthostatic intolerance and controls, respectively were 20.5, 19.5, 6.0).

## Discussion

We used a simple, real-life orthostatic challenge to determine rates of the different physiological manifestations of orthostatic intolerance in CFS. Previous studies of orthostatic intolerance in CFS have focused on changes in blood pressure and heart rate with approximately 25% of patients having these abnormalities. [19]. However, orthostatic changes in heart rate and blood pressure are not uncommon in healthy people too [4,11]. In our studies, CFS patients did show higher rates of orthostatic tachycardia, hypertension, and hypotension than healthy controls. However, the differences were not significant, and substantially larger sample sizes would have been necessary for significance to have emerged.

In contrast, 21% of CFS patients studied here compared to only 3% of controls had orthostatic hypocapnia, usually occurring without cardiovascular indices of OI. These patients reported more problems with shortness of breath and feeling ill during the orthostatic challenge than patients without physiological evidence of OI or controls. An earlier study using a longer duration orthostatic challenge – 30 min of head up tilt – noted hypocapnia to occur in the presence of other cardiovascular indices of OI [9]. That report as well as this one suggests that alterations in respiration are the primary manifestation of OI in patients with CFS. The identification of a subset of CFS patients with this physiological manifestation of orthostatic intolerance is important in that its existence can be used as a stratification strategy to reduce the patient pool heterogeneity inherent in using a clinical case definition to diagnose CFS.

We thought we might find a relation between CFS illness severity and orthostatic intolerance, but we did not. We found the same rates of "severe CFS" in patients with orthostatic hypocapnia as in patients without orthostatic intolerance. In addition, we found no difference in rates of clinically meaningful depression in the two CFS groups as assessed by the CES-D. Whether some other illness-related variable is predictive of orthostatic intolerance remains to be determined.

One limitation in our study was that we evaluated successive patients in either a private practice or a research setting regardless of whether or not they were taking medicine. While we did drop data from two patients who developed orthostatic hypotension due to their being on anti-hypertensive medication, use of other medications did not explain the tendency of patients to show more orthostatic hypotension or tachycardia than controls. It is not apparent why medications would produce orthostatic hypocapnia in the absence of other syndromes of orthostatic intolerance; however, this remains a possibility which will require further study of unmedicated CFS patients.

There are at least two explanations to account for orthostatic hypocapnia – hyperventilation or reduced delivery of CO2 to the lung secondary to reduced venous return to the right side of the heart. If it were the latter, one would expect transient hypocapnia occurring early during standing. Instead, we found that hypocapnia was sustained and progressive, and the hypocapnia usually occurred without other cardiovascular manifestations of orthostatic intolerance. This analysis supports the idea that the hypocapnia was due to hyperventilation; although we did not assess ventilation in this study, we did in another study and found hyperventilation in adolescents who became hypocapnic during tilt testing [20]. But why orthostatic hypocapnia develops as the primary mechanism for developing symptoms of OI in CFS patients is an important research question. Our data indicate that emotional factors related to anxiety or depression are not important. Our working hypothesis is that this phenomenon comes from a complex interaction among the baroreflex, chemoreceptors, and thoracic blood volume. Nonetheless, the occurrence of isolated orthostatic hypocapnia in CFS suggests that it is an important marker for orthostatic intolerance in some patients with medically unexplained fatigue, which may eventually be susceptible to treatment. Finding such a marker in a subgroup of CFS patients is the first step in moving this illness from a clinical syndrome to one diagnosable by laboratory testing.

## Conclusion

The occurrence of isolated orthostatic hypocapnia in CFS suggests that it is an important marker for orthostatic intolerance in some patients with medically unexplained fatigue, which may eventually be susceptible to treatment. Finding such a marker in a subgroup of CFS patients is the first step in reducing the patient pool heterogeneity implicit in using a clinical case definition for diagnosis.
